# Dorzolamide Loaded Niosomal Vesicles: Comparison of Passive and Remote Loading Methods

**Published:** 2017

**Authors:** Mohadeseh Hashemi Dehaghi, Azadeh Haeri, Hamid Keshvari, Zahra Abbasian, Simin Dadashzadeh

**Affiliations:** a *Department of Pharmaceutics, School of Pharmacy, Shahid Beheshti University of Medical Science, Tehran, Iran.*; b *Department of Biomedical Engineering, Faculty of New Science and Technology, Tehran University, Tehran, Iran.*; c *Department of Biomedical Engineering, Amir Kabir University of Technology, Tehran, Iran.*

**Keywords:** Niosome, glaucoma, dorzolamide, thin film hydration method, phosphate gradient method

## Abstract

Glaucoma is a common progressive eye disorder and the treatment strategies will benefit from nanoparticulate delivery systems with high drug loading and sustained delivery of intraocular pressure lowering agents. Niosomes have been reported as a novel approach to improve drug low corneal penetration and bioavailability characteristics. Along with this, poor entrapment efficiency of hydrophilic drug in niosomal formulation remains as a major formulation challenge. Taking this perspective into consideration, dorzolamide niosomes were prepared employing two different loading methodologies (passive and remote loading methods) and the effects of various formulation variables (lipid to drug ratio, cholesterol percentage, drug concentration, freeze/thaw cycles, TPGS content, and external and internal buffer molarity and pH) on encapsulation efficiency were assessed. Encapsulation of dorzolamide within niosomes increased remarkably by the incorporation of higher cholesterol percentage as well as increasing the total lipid concentration. Remote loading method showed higher efficacy for drug entrapment compared to passive loading technique. Incorporation of TPGS in bilayer led to decrease in EE; however, retarded drug release rate. Scanning electron microscopy (SEM) studies confirmed homogeneous particle distribution, and spherical shape with smooth surface. In conclusion, the highest encapsulation can be obtained using phosphate gradient method and 50% cholesterol in Span 60 niosomal formulation.

## Introduction

Glaucoma is a progressive optic neuropathy which is characterized by an increase in the intraocular pressure (IOP) following damage to ganglionic cells and the optic nerve (1, 2). The rise of IOP associated with glaucoma is caused by the imbalance between the aqueous humor fluid production in the eye and its outflow through the trabecular meshwork in Schlemm’s canal (3). Medications are prescribed to decrease the amount of aqueous humor produced by the ciliary body or to increase its outflow through the trabecular meshwork, through the uveoscleral pathway, or through a surgically created pathway (2, 4). 

Clinical experiments suggest that the conventional methods of treating glaucoma (ocular drops) are associated with poor bioavailability of the active ingredients to the ophthalmic receptors. The causes of poor ocular drug bioavailability (< 1%) from conventional eye drops are high tear fluid turnover, nonproductive absorption, unstable residence in the cul-de-sac, and the relative impermeability of the drugs to corneal epithelial membrane (5, 6).

Various researches have been performed to overcome the disadvantages of conventional glaucoma treatments (6, 7). Among them, colloidal drug delivery systems including liposomes (8, 9), niosomes (10), and polymeric nanoparticles (11), can increase ocular bioavailability of administered drugs. Most of these ophthalmic delivery systems can prolong and control drug actions at the corneal surface and prevent enzymatic drug metabolism (6, 7). Moreover, the presence of surfactant and cosurfactant could enhance drug permeability (12-14).

Niosomes and liposomes are two versatile bilayer vesicles made of non-ionic surfactants and phospholipids, respectively (15, 16). Although there is a great interest in liposomes, they have limitations such as chemical instability due to the oxidative degradation, limited shelf life, phospholipid impurity, and high cost (15). Niosomes have been investigated as an alternative to liposomes. Niosomes are defined as microscopic lamellar structures obtained during hydration of nonionic surfactants and cholesterol mixture (17, 18) and proposed as a novel carrier for ocular delivery (19, 20). 

Dorzolamide HCl is a carbonic anhydrase inhibitor, which is administered for open angle glaucoma. Its dosage form is 0.2% w/v (2 mg/mL) eye drop that is administered 2-3 times daily. Tear turnover, blinking, and enzymatic degradation in tear fluid influence the drug efficiency to the extent that only a few percentages of the instilled drug become available for absorption (21, 22).

The objective of the present study was to encapsulate dorzolamide HCl in niosomal formulation for ocular delivery. In the current study, the niosomal vesicles were formulated by both thin film hydration and phosphate gradient methods. The latter was reported for encapsulation of doxorubicin in liposome by Fritze *et al.* (23). In current study, many factors that may influence drug entrapment have been examined and optimized. To the best of our knowledge, the present study is the first report on using phosphate gradient method to encapsulate drugs in niosomal vesicles and also studying the effect of d-α-tocopheryl polyethylene glycol 1000 succinate (TPGS) incorporation in niosomal formulations.

## Materials and methods

*Materials*

Dorzolamide HCl was kindly donated by Sina-Darou Pharmaceutical Company, Tehran, Iran. Sorbitan monostearate (Span 60), cholesterol (99%), and d-α-tocopheryl polyethylene glycol 1000 succinate (TPGS) were all purchased from Sigma–Aldrich, UK. Di-ammonium hydrogen phosphate salts, chloroform, hydrochloric acid, 2-propanol, 4-(2-hydroxyethyl)-piperazine]-ethanesulfonic acid (HEPES), di-sodium hydrogen phosphate heptahydrate (Na_2_HPO_4_. 7H_2_O), potassium dihydrogen phosphate (KH_2_PO_4_), potassium chloride, and sodium chloride were all supplied by Merck, Germany. Cellulose dialysis tubing (molecular weight cutoff 12,000 Da) was purchased from BioGene, USA.

*Niosome preparation*

*Thin film formation*

Nonionic surfactant (Span 60) and cholesterol were accurately weighed, and dissolved in 5 mL chloroform, and the solvent was evaporated under reduced pressure using a rotary evaporator at 65 °C to form thin lipid film on the inner flask wall. Evaporation was continued for 2 h to remove residual solvent (24).

*Drug loading process*

In order to encapsulate dorzolamide in niosomal formulations two methods of thin film hydration, as a passive loading method, and phosphate gradient, as a remote loading method, were investigated.

*Passive loading method*

Niosomes containing dorzolamide were prepared by thin film hydration method, as described previously by Baillie *et al*. (25). After thin film formation, as mentioned above, the dried film was then hydrated by desired pre-heated amount of phosphate buffer saline containing various concentration of drug at 65 °C for 1.5 h. During hydration step, niosomal suspensions were sonicated in a bath sonicator to reduce the size. Formulations were allowed to anneal and stabilize for 1 h at the room temperature and then were left to mature overnight at 4 °C. 

*Phosphate gradient (remote) loading method*

To encapsulation of dorzolamide using phosphate gradient method (23), thin film was formed as mentioned above, and the film was then hydrated using di-ammonium hydrogen phosphate with same condition as above mentioned for the passive method. Empty niosomal suspensions were sealed in dialysis bag (MW: 12,000 Da) and immersed into an isotonic HEPES buffered saline (HBS), 140 mM NaCl, 10 mM HEPES to replace the extra-niosomal buffer. The process was continued for 4 h. Subsequently and also the desired amount of dorzolamide HCl was added to the niosomal dispersion. The loading process was carried out at 60-65 °C for 1 h. 

*Freeze/thaw process of formulations*

To study the effect of freeze/thaw cycles on EE (%), each dispersion (2 mL) was frozen for 5 min at -196 °C in liquid nitrogen bath and then thawed for 5 min at 65 °C in a water bath, which caused the lipid bilayer to break upon cooling and reform upon heating.

*Niosome characterization*

The prepared niosomes were characterized regarding encapsulation efficiency (EE %), size, morphology, and in vitro drug release profile. 

*Determination of dorzolamide entrapment efficiency*

Vesicles containing dorzolamide were separated from unencapsulated drug by dialysis bag. Niosomal suspensions were placed in the dialysis bag which was then placed in beaker containing phosphate-buffered saline (PBS, pH 7.4) with volume more than 100 times greater than inner bag volume with constant stirring for 1 h in 4 °C. For vesicle digestion, 2-propanol was added, and then the suspensions were sonicated in a bath sonicator for 10 min. The solutions were measured spectrophotometrically at 254 nm and dorzolamide concentration was determined.

The EE was calculated as follow:

EE (%) = (amount of dorzolamide encapsulated)/(total amount of dorzolamide)

*In-vitro release profile*

The in vitro release of dorzolamide HCl from niosomal formulation was determined by membrane diffusion method (24, 26). In brief, the presoaked dialysis bag containing 0.5 mL of the niosomal formulations was suspended in the beaker containing 20 mL of PBS (pH 7.4) and stirred (200 rpm) at 37 ± 0.5 °C. At predetermined time points, desired amount of the sample was withdrawn from the beaker and analyzed by UV spectrophotometer.

*Size and morphology characterization*

The morphology, size, and size distribution of niosomal vesicles were analyzed by field emission scanning electron microscopy (SEM, model S4160, Hitachi) and dynamic light scattering after appropriate dilution. 

*Statistical Analysis*

The results were analyzed by one-way analysis of variance (ANOVA) followed by Tukey’s pairwise test at 5% significance level. P values< 0.05 were considered significant.

## Results and Discussion

*Thin film hydration (passive) method*

**Figure 1:**
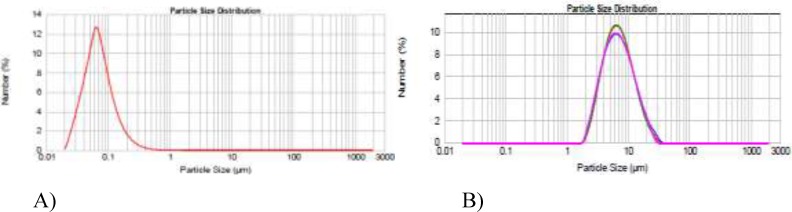
The effect of rotational speed on nanoparticles size, A) 150 rpm and B) 60 rpm

Thin film hydration is the most simple, repeatable, and extensively studied method to prepare multilayer vesicles (MLV). To optimize the niosomal formulations with regard to size, two formulations were prepared at the rotary evaporator rotational speed of either 60 rpm or 150 rpm and the vesicular size was examined by dynamic light scattering (Figure 1). As shown in Figure 1 by increasing the rotational speed from 60 to 150 rpm, the average particle sizes decreased from 800 to 150 nm. Furthermore, different periods of hydration time (0.5, 1, 1.5, and 2 h) were investigated to improve EE. EE remained unchanged regardless of hydration time (data not shown).

**Table 1:** Influence of formulation variables on EE (%) of niosomes prepared by thin-film hydration method (n = 3, mean ± SD

**Formula**	**L/D**	**Span/cholesterol**	**Drug Concentration (mg/mL)**	**EE (%)**
**F1**	10	70/30	0.5	1.8 ± 0.3
**F2**	20	70/30	0.5	5.6 ± 0.6
**F3**	30	70/30	0.5	23.5 ± 0.5
**F5**	40	70/30	0.5	24.5 ± 0.7
**F6**	30	80/20	0.5	18.1 ± 0.8
**F7**	30	60/40	0.5	27.5 ± 0.5
**F8**	30	50/50	0.5	24.3 ± 0.7
**F9**	30	60/40	1	20.2 ± 0.9
**F10**	30	60/40	2.5	7.7 ± 2.0

**Table 2:** Influence of freeze thaw cycles on EE (%) of niosomes prepared by thin-film hydration method (n = 3, mean ± SD

**Formula**	**L/D**	**Span/cholesterol**	**Drug Concentration (mg/mL)**	**Number of freeze–thaw** **cycles**	**EE (%)**
**F3**	30	70/30	0.5	0	23.5 ± 0.5
**F11**	30	70/30	0.5	1	28.3 ± 1.5
**F12**	30	70/30	0.5	2	28.3 ± 0.8
**F13**	30	70/30	0.5	3	16.4 ± 0.6
**F14**	30	70/30	0.5	4	13.0 ± 0.7

**Table 3:** Influence of formulation variables on EE (%) of niosomes prepared by remote loading method (n = 3, mean ± SD).

**Formula**	**di-Ammonium hydrogen phosphate's molarity(mM)**	**di-Ammonium hydrogen phosphate's pH**	**pH of Hepes-Saline**	**L/D**	**Span/ ** **cholesterol**	**Drug ** **concentration** **(mg/mL)**	**EE (%)**
**F16**	200	6.5	7.8	10	60/40	0.5	32.1 ± 1.3
**F17**	250	6.5	7.8	10	60/40	0.5	NF*
**F18**	300	6.5	7.8	10	60/40	0.5	NF*
**F19**	200	5.5	7.8	10	60/40	0.5	NF*
**F20**	200	6	7.8	10	60/40	0.5	NF*
**F21**	200	6.5	7.3	10	60/40	0.5	31.2 ± 1.0
**F22**	200	6.5	8.3	10	60/40	0.5	31.6 ± 1.6
**F23**	200	6.5	7.8	15	60/40	0.5	36.0 ± 0.6
**F24**	200	6.5	7.8	20	60/40	0.5	NF*
**F25**	200	6.5	7.8	15	50/50	0.5	39.6 ± 0.4
**F26**	200	6.5	7.8	15	60/40	2	47.7 ± 1.3

* Vesicles were not formed.

**Figure 2:**
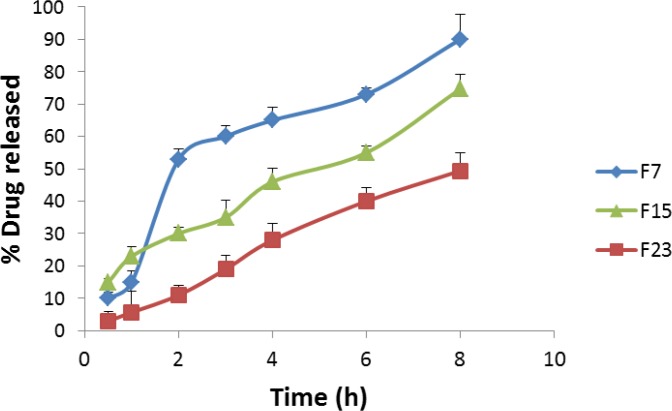
Effect of TPGS content and niosomal preparation method on the in vitro release of dorzolamide from vesicles. F7: formulation prepared by passive loading method; F15: formulation containing 10% TPGS prepared by passive loading method; F23: formulation prepared by remote loading method

**Figure 3:**
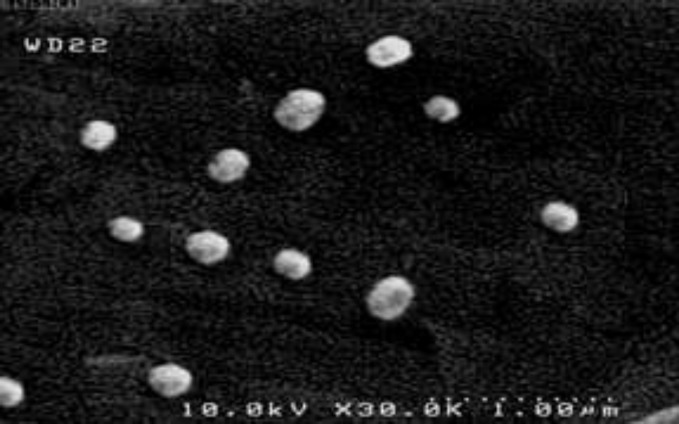
Scanning electron microscopy (SEM) micrograph of niosomes composed of Span 60 and cholesterol in 60: 40 molar ratio

prepared by transmembrane phosphate gradient.

The influence of some important variables including lipid to drug ratio (L/D), cholesterol percentage, drug concentration in hydration medium, the number of freeze/thaw cycles, and TPGS content on EE (%) of dorzolamide HCl in niosomal formulation was investigated.

*Effect of lipid to drug ratio (L/D)*

To investigate the influence of lipid to drug ratio on EE (%), different L/D ratios (10, 20, 30, and 40) were examined. As shown in Table 1, dorzolamide entrapment efficiency increased from 1.8 ± 0.3 to 23.5 ± 0.5%, by increasing L/D from 10 to 30 (P ˂ 0.001), however, by increasing L/D from 30 to 40, EE did not significantly increase. Similar results were obtained by Dadashzadeh and her coworkers, reported an increase in the EE of hydrophilic and hydrophobic drugs with increasing total lipid concentration (27-30). Although increasing the total lipid level causes increase in the EE of drug molecules, in some cases, it leads to increase in system viscosity which is not desirable. Further increase of lipid/drug molar ratio was not investigated because the amount of lipid and surfactant that could be administered for a given drug dose is limited. High lipid and surfactant doses may raise concerns of toxicity, reduce the economic feasibility of pharmaceutical scale production, and worsen the physical characteristics of the dosage form.

*Effect of Span to cholesterol ratio*

In order to find the role of cholesterol content on EE, various ratios of Span 60 to cholesterol were examined (Table 1, F3, F6, F7, and F8). When cholesterol molar ratio increased from 10% to 40%, the EE (%) significantly increased from 18.1 ± 0.8 to 27.5 ± 0.5 (P ˂ 0.001). Similar results were obtained for encapsulation of anthracyclines into liposomes (31). Increase in the cholesterol content of the bilayer resulted in increase of the bilayer rigidity, higher stability, and reduced permeability of the system (32), and hence increased drug retention into the niosomal vesicles. However, when amounts of cholesterol increased further from 40 to 50% molar ratio, the opposite result was observed (Table 1). This could be due to the fact that the cholesterol amounts in a certain level start distorting the bilayer structure leading to loss of entrapped drug (33). Therefore, at this step, 40% cholesterol was chosen as the optimum content.

*Effect of drug concentration*

We hypothesized that the saturation and the hydration media with drug can push the drug to be encapsulated within the vesicular system as previously reported by EL-Samaligy *et al. *(34) and Mokhtar *et al.* (35). To determine the influence of drug concentration on EE (%), various dorzolamide concentrations (0.5, 1, 2.5 mg/mL) were studied (Table 1). It was observed that by increase in the drug concentration from 0.5 to 2.5 mg/mL, the drug EE (%) decreased from 27.5 ± 0.5 to 7.7 ± 2.0% (P ˂ 0.001). 

*Effect of freeze/thaw cycles*

To study the effect of freeze/thaw cycles on EE (%), after niosomal vesicles formation by thin film hydration method, each dispersion (2 mL) was frozen for 5 min at -196 °C in liquid nitrogen bath and then thawed for 5 min at 65 °C in a water bath, which caused the lipid bilayer to break upon cooling and reform upon heating. The cycle was repeated 1 to 4 times (Table 2).

This study was performed on the formulations containing 30% cholesterol, because the increase in cholesterol percentage could lead to increase in the rigidity of the bilayer and as a result led to disrupting the bilayer during freeze-thaw procedure. As shown in Table 2, the EE (%) of the niosomal formulation increased from 23.5 ± 0.5 to 28.3 ± 1.5% during freeze-thaw process. In second freeze-thaw cycle, the EE (%) was not changed. But, after the third freeze-thaw cycle, the EE (%) was significantly decreased (P < 0.001). Zhao and Lu reported increase in the EE (%) of the encapsulated drug during freeze-thaw cycles (36). Similar result was reported by Buchanan et al suggesting that, during disruption and fusion occurred in freeze-thaw procedure, the EE (%) of the NF-κB decoy oligonucleotides increased (37). In this study marginal improvement in EE was observed.

*Effect of TPGS content*

Vitamin E TPGS is a surfactant that has been used as a drug solubilizer, emulsifier, absorption enhancer, and as a vehicle for drug-delivery (38). Using TPGS as a coating material on the liposomal vehicle was studied to enhance cellular uptake and target drug delivery. This surfactant, with high HLB value related to large head group, was suggested to be a good vehicle for encapsulation of hydrophilic drugs like dorzolamide HCl, and using TPGS as a part of niosomal vesicles was reported in this study for the first time. 

By incorporation of 10% TPGS content in niosomal formulations (F15), the EE (%) of dorzolamide non-significantly decreased from 27.5 ± 0.5 to 26.5 ± 1.7%.

*Phosphate gradient (remote) method*

According to the previous section, the maximum EE (%) achieved by thin film hydration method was ~28%. Although thin-film hydration is a simple, repeatable, and most studied technique, one of the disadvantages of this method is its relatively poor EE (5-15%) for hydrophilic drugs, like dorzolamide HCl. In other studies, brimonidine tartrate and acetazolamide as anti-glaucoma drugs were encapsulated in niosomal vesicle by thin film hydration method, and the maximum EE was reported to be 32.27% and 32.21%, respectively (39, 40).

Compared with passive loading, pH gradient method possesses the advantage of high EE and high drug loading rate (31). One of the commercial liposomal formulation that encapsulated by pH gradient was Myocet^TM^ (liposomal doxorubicin) with EE above 95% (31).

Niosomal preparation by using remote loading (i.e. pH gradient method) may have higher EE than passive loading (i.e. thin film hydration method). As a similar result, urea gel was encapsulated in niosomes by using both thin film hydration and pH gradient methods, maximum EE(%) values were 13.4 ± 1.2% and 52.9 ± 2.3%, respectively (41). The phosphate gradient method was first introduced by Fritze et al. for encapsulating doxorubicin HCl in liposomal vesicles and was reported to be more efficient than pH gradient method (23). In the present study, dorzolamide HCl was encapsulated in niosomal vesicles by using phosphate gradient method.

To optimize niosomal formulation prepared by phosphate gradient method, some important factors were evaluated including intravesicular phosphate concentration, interior and exterior pH values, L/D, Span 60 to cholesterol ratio, drug concentration, and TPGS (%) content.

*Effect of intravesicular phosphate concentration*

For studying the effect of intravesicular phosphate concentration, di-ammonium hydrogen phosphate with different concentrations of 200, 250, and 300 mM (pH 6.5) was used as hydration medium (F16, F17, and F18, Table 3). We expected that as the molarity of di-ammonium hydrogen phosphate increased, the EE (%) of dorzolamide HCl increased, but as shown in Table 3, in 250 and 300 mM, no vesicle was formed.

*Effect of different interior pH values*

To investigate the effect of the interior pH, 200 mM di-ammonium hydrogen phosphate with different pH values (5.5, 6, and 6.5) was used as an interior buffer during hydration process (F16, F19, and F20, Table 3). We expected that by increasing the pH gradient, the EE (%) of dorzolamide HCl would improve, but as shown in Table 3 in pH values of 5.5 and 6, stable vesicle was not formed.

*Effect of different exterior pH values*

The effect of exterior pH values on the drug EE (%) was investigated by using HEPES buffer saline with different pH values as external buffer (F16, F21, and F22). But as data shown in Table 3, changing external pH did not have significant effect on EE (%) (P ˃ 0.05). 

*Effect of L/D*

To investigate the effect of lipid to drug ratio on remote loading method, formulations with L/D = 10, 15, and 20 were prepared. By increasing lipid to drug ratio from 10 to 15, EE (%) increased from 32 to 36 (F16 vs. F23) (P = 0.029). In L/D = 20, stable vesicle was not formed. The effect of lipid to drug ratio on concentration of vesicles was discussed previously (Section 3.1.1) for passive loading and similar results were observed for remote loading. In remote loading with L/D of 15 which was half of the used L/D in passive loading, we achieved higher EE.

*Effect of Span 60 to cholesterol ratio*

Similar to passive loading, to investigate the effect of Span 60 to cholesterol ratio on achieved EE (%) in remote loading method, formulations with Span/cholesterol of 60/40 and 50/50 were prepared and characterized. When cholesterol molar ratio increased from 40% to 50%, the EE significantly improved from 36.0 ± 0.6% to 39.6 ± 0.4% as shown in Table 3.

*Effect of drug concentration*

As shown in Table 3, by increasing drug concentration from 0.5 to 2 mg/mL, (F25 and F26), the EE (%) of dorzolamide increased from 36 to 48 (P ˂ 0.001). This was in contrary with the observed results in thin film hydration method (Section 3.1.3).

*Effect of TPGS content*

Similar to thin film hydration method incorporation of 10% TPGS to bilayer composition resulted in significant decrease in EE (P ˂ 0.001). As the TPGS content increased from 0 to 10%, EE (%) significantly decreased from 47.7 ± 1.4% to 19.8 ± 0.9%.

*In-vitro release*

Besides efficient loading, the drug release rate is also a critical factor for a drug delivery system. Controlling release of the drug from vesicles is really important, because prolonging drug retention on the eye surface could lead to increase drug bioavailability and therapeutic effect. The effect of TPGS content on drug release was studied. Moreover, *in-v*itro drug release from the niosomal formulation prepared by thin film hydration and phosphate gradient methods was examined. As shown in Figure 2, by incorporating 10% TPGS in niosomal formulations, the rates of drug release decreased significantly. After 8 hour the amounts of drug release from formulation without TPGS was 90.3 ± 7.6%, however incorporating 10% TPGS decreased amount of drug release to 74.7 ± 4.6%. It is assumed that the TPGS surfactant acts as a steric stabilizer at the niosomal bilayer.

Drug release from the formulation prepared by phosphate gradient method was slower than the one prepared by thin film hydration method (Figure 2). In niosomal formulation prepared by thin film hydration, after 1 hour 14.8 ± 3.6% of drug released from formulation, while in phosphate gradient method, the amounts of released drug after 1 hour was 5.7 ± 6.4%. As data shown, after 8 hours the amounts of drug released from thin film hydration method and phosphate gradient method were 90.3 ± 7.6 and 49.4 ± 5.4%, respectively. Two synergistic effects may play roles in loading of dorzolamide by means of the salt gradient, both of which could result in the fact that drug molecules pass the lipid membrane slower. On the one hand, loading is driven by protonation and charging of drug within the niosomes; on the other hand, it is driven by precipitation of dorzolamide in the hydrophilic interior of the vesicle when the drug concentration exceeds its solubility. These explanations were also reported for drug entrapped liposomes prepared by remote loading methods (23, 31). 

*Scanning electron microscope (SEM) characterization*

The surface morphology of the niosomes composed of Span 60 and cholesterol in 60: 40 molar ratio prepared by transmembrane phosphate gradient method was shown in Figure 3. The prepared nanoparticles were spherical in shape and uniform in size. Particle size ranged from 150 to 300 nm.

## Conclusion

In the present study, the effect of variable parameters on EE of dorzolamide HCl in passive loading (thin film hydration method) and remote loading (phosphate gradient method) was investigated for the first time. In thin film hydration method, L/D, cholesterol content, drug concentration, number of freeze/thaw cycles, and TPGS incorporation were studied. In phosphate gradient method, intravesicular phosphate concentration, interior and exterior pH values, L/D, total cholesterol amount, drug concentration, and TPGS (%) were analyzed. The results showed maximum dorzolamide encapsulated in niosomal formulation prepared by thin film hydration and phosphate gradient methods were 28.3 ± 1.5% and 47.7 ± 1.3%, respectively.
